# Comment on Moz-Christofoletti, M.A.; Wollgast, J. Sugars, Salt, Saturated Fat and Fibre Purchased through Packaged Food and Soft Drinks in Europe 2015–2018: Are We Making Progress? *Nutrients* 2021, *13*, 2416

**DOI:** 10.3390/nu14051116

**Published:** 2022-03-07

**Authors:** Nicholas Hodac, Anna Wittekind

**Affiliations:** 1 UNESDA, Rue du Trône 14-16, 1000 Brussels, Belgium; 2 Independent Researcher, Buckinghamshire HP16 0BS, UK; anna@awnutrition.co.uk

The European soft drinks industry fully supports efforts to monitor nutrition composition of food products. We were therefore interested to read the European Commission’s Joint Research Centre (JRC) publication by Moz-Christofoletti and Wollgast, which attempted to assess progress on processed foods versus existing commitments across European countries between 2015 and 2018 [1].

The rationale for the study was based firstly on a recognition that monitoring activities are limited by lack of a harmonised system, with studies employing different methodologies, time frames and product categorisations, and secondly that the Europe-wide Joint Action Best-ReMaP project [2], which is developing a European standardised monitoring system to try and address these limitations, will take time to produce results.

Given the JRC’s remit to provide independent scientific advice to support EU policy, it is vital that progress which has been achieved is not undermined by analyses being subject to some of the aforementioned limitations, in this case by only including retail sales and using product category definitions which are not in line with those of the EU.

Although the paper provides definitions for the sub-categories of products included in each of the food and beverage categories in an Appendix [1], it may not be sufficiently clear to the reader that their “Soft Drinks” category included 100% juice—the latter is a separate category under EU food law which cannot legally be allowed to be reformulated and still sold as juice. While we understand that inclusion of 100% juice has been driven by the product categories of the commercial provider of the data, this inclusion has severely diluted the relative progress which has been achieved in sugar reduction using the EU definition of soft drinks.

In addition, the maximum levels of total sugars (g/100 mL) for soft drinks in Table 2 [1], appear to include concentrates (liquids/powders) which, without application of a dilution factor that is always applied when consumed, result in exceedingly high sugar levels (>90 g/100 mL).

The authors briefly discussed various limitations of their approach but given the significance of their suggestion that we are falling short in our progress against commitments, we feel they should have more clearly identified and discussed such limitations. UNESDA’s own data from a similar commercial provider estimated that the European soft drink industry has achieved a 14.6% reduction in added sugars from 2015 to 2019 [3], exceeding the pledge to the EU Annex on Added Sugars [4].

While we appreciate that all data, from whatever source, has limitations, we would kindly request that the limitations in this case are clearly understood.
